# Quality of life and burden of caregivers of patients with alpha-mannosidosis: an international survey

**DOI:** 10.1186/s13023-026-04496-y

**Published:** 2026-07-22

**Authors:** Julia B. Hennermann, Nathalie Guffon, Sophie Thomas, Karolina M. Stepien, Christina Lampe, Nicole M. Muschol, Maria Juliana Ballesta-Martínez, Jordi Cruz, Mónica López-Rodríguez, Anneliese Barth, Allan M. Lund, Vasilica Plaiasu, Chiara Donati, Heather M. Morgan, Andrea Ballabeni, Martin Magner

**Affiliations:** 1 Villa Metabolica, University Medical Center Mainz, Mainz, Germany; 2 Reference Center for Inherited Metabolic Disorders, Femme Mère Enfant Hospital, Hospices Civils de Lyon, Lyon, France; 3 The Society for Mucopolysaccharide and Related Diseases, MPS House, Amersham, UK; 4 Adult Inherited Metabolic Disorders, Salford Royal Hospital, Northern Care Alliance NHS Foundation Trust, Salford, UK; 5 Centre for Rare Diseases, University Hospital of Giessen, Giessen, Germany; 6 International Center for Lysosomal Disorders (ICLD), University Medical Center Hamburg- Eppendorf, Hamburg, Germany; 7 Sección de Genética Médica, Hospital Universitario Virgen de la Arrixaca, Murcia, Spain; 8 Asociación MPS-Lisosomales España, Barcelona, Spain; 9 Hospital Universitario Ramón y Cajal, IRYCIS, Madrid, Spain; 10 Instituto Nacional de Saúde da Mulher, da Criança e do Adolescente Fernandes Figueira - IFF/Fiocruz, Rio de Janeiro, Brazil; 11 Centre for Inherited Metabolic Diseases, Copenhagen University Hospital, Copenhagen, Denmark; 12 Regional Center of Medical Genetics Bucharest, INSMC Alessandrescu-Rusescu, Bucharest, Romania; 13 Chiesi Farmaceutici S.p.A, Parma, Italy; 14 Chiesi USA Inc., Boston, USA; 15 Department of Pediatrics and Inherited Metabolic Disorders, General University Hospital and First Faculty of Medicine, Charles University, Prague, Czech Republic

**Keywords:** Alpha-mannosidosis, Caregivers, Quality of life, Survey, Mental health

## Abstract

**Background:**

This study assessed quality of life (QoL) changes over time amongst caregivers of patients with alpha-mannosidosis, a rare autosomal recessive lysosomal storage disorder with progressive multi-systemic effects. An international online survey was distributed to caregivers of patients aged ≥ 10 years, including visual analogue scales (VAS; timepoints 5 years prior and current) and multiple choice and open text questions. Survey questions related to five QoL domains (physical health, mental health, family and social life, relationships with partners/family/friends and ability to work/attend education).

**Results:**

Forty-three caregiver responses from 16 countries were analysed. Caregivers looked after 43 patients: 16 untreated patients (UP), six who had undergone allogenic haematopoietic stem cell transplantation (HSCT) and 21 receiving enzyme replacement therapy (ERT). From 5 years prior to the time of survey participation, physical health slightly improved for caregivers of ERT and HSCT patients (mean ± standard deviation changes in VAS − 0.1 ± 2.8 and − 0.2 ± 1.2, respectively) but slightly declined among UP caregivers (+ 0.7 ± 0.9). Mental health improved for caregivers of HSCT patients (-1.0 ± 4.7) but stabilised and worsened for ERT and UP caregivers, respectively (ERT: 0.0 ± 2.3; UP: +1.4 ± 1.9). Family and social life scores improved among caregivers of HSCT patients (-1.0 ± 3.5) but slightly declined and worsened for ERT (+ 0.1 ± 2.3) and UP (+ 1.1 ± 1.4) caregivers, respectively. Relationship scores slightly improved for caregivers of ERT and HSCT patients (both − 0.2) but worsened among UP caregivers (+ 1.3 ± 1.7). There were slight improvements in ability to work or attend education for caregivers of ERT and HSCT patients.

**Conclusions:**

Although a causal relationship between disease-modifying treatment and caregiver QoL could not be determined, mostly due to small subgroup sizes and age differences between HSCT patients, ERT patients and UP, caregivers of patients receiving ERT or HSCT generally experienced stable or improved/slightly improved QoL outcomes over the 5-year study period. In contrast, caregivers of UP consistently reported deterioration across physical, mental, social and relational QoL domains. These patterns suggest that access to disease-modifying treatment could help mitigate declines in caregiver QoL, highlighting the need to routinely assess caregiver-reported outcomes and support caregiver needs alongside patient management in alpha-mannosidosis.

**Supplementary Information:**

The online version contains supplementary material available at https://doi.org/10.1186/s13023-026-04496-y.

## Background

Alpha-mannosidosis (OMIM 248500), a rare progressive autosomal recessive inherited lysosomal storage disorder (LSD) caused by pathogenic variants in the *MAN2B1* gene (OMIM 609458), leads to deficient activity of the alpha-mannosidase enzyme and accumulation of undegraded mannose-rich oligosaccharides in cells [1–4]. Its prevalence is estimated at 1:300,000 to 1:1,000,000 live births [3]. Most children present with clinical manifestations in their first decade of life, which progressively worsen over time [4, 5]. Symptoms include facial and skeletal abnormalities, hearing loss, speech difficulties, immunodeficiency with recurrent infections, muscular weakness and ataxia, cognitive impairment and psychiatric manifestations [4–8]. Attenuated forms are associated with slow progression and survival into adulthood; severe forms with rapid progression and death in childhood [3]. Disease specific treatments for alpha-mannosidosis are limited. Allogenic haematopoietic stem cell transplantation (HSCT) may be effective in improving physical manifestations and in preserving neurocognitive function and has shown promising results in some patients [3, 9, 10]. A clinical study of 21 children with alpha-mannosidosis concluded that HSCT was associated with clinical benefit compared with pre-HSCT findings, with acceptable safety outcomes [10]. HSCT should be conducted as early as possible to prevent irreversible pathological changes [3]; however, there are associated morbidity and mortality risks [3]. Alternative disease-modifying approaches have therefore been explored. The first human recombinant form of alpha-mannosidase available for enzyme replacement therapy (ERT), velmanase alfa, significantly reduced serum oligosaccharide concentration in placebo-controlled studies; trends towards improvement were observed in immune, motor and lung function and quality of life (QoL) in placebo-controlled and open-label studies [11–13]. A long-term (12 year) follow-up efficacy and safety study has since demonstrated continued benefits of velmanase alfa in patients with alpha-mannosidosis [14]. However, despite the availability of disease-modifying therapies, not all patients receive such treatment. Some individuals remain untreated and instead receive supportive care alone [15]; these patients are referred to as untreated patients (UP). The rarity and the heterogeneous presentation of alpha-mannosidosis may lead to long delays between symptom onset and diagnosis, which contribute to patients’ clinical deterioration [16]. It has been described that delayed diagnoses as well as a lack of information on the expected progression and future outcomes of rare diseases can cause considerable stress and anxiety for caregivers [17, 18]. Caregivers of patients with rare diseases experience reduced QoL, not only due to concerns for the patient, but also due to impairment of their own physical and mental health, juggling employment or educational commitments and facing the financial demands of caregiving [17, 19, 20]. Similar to other LSDs, the heterogeneity of alpha-mannosidosis symptoms, disease severity and progression rates can contribute to a significant burden on caregivers and family members. Patients with alpha-mannosidosis often experience a distinctive combination of cognitive, behavioural, hearing and musculoskeletal symptoms [3, 4], which is likely to lead to a uniquely high practical, physical and emotional burden for caregivers and family members. Moreover, although patients with alpha-mannosidosis are typically cared for by a family member, some may require long-term residential care, for example, when delayed diagnosis and access to treatment have resulted in cumulative morbidity [21]. Although a substantial caregiver burden has been described for LSDs [17, 18, 21–25], data on the experiences of caregivers of patients with alpha-mannosidosis remain limited [21, 22]. The benefit of HSCT or ERT on caregiver QoL has been described for just four caregivers in the UK [22]. It is therefore vital to further assess the experiences of individuals providing care in various alpha-mannosidosis disease trajectories, as their perspective provides valuable insight into the long-term impact of disease progression on caregivers and families. Recognising the challenges these caregivers face can enable the provision of targeted support, helping caregivers safeguard their own health and well-being while continuing to provide optimal care and reducing the broader social and economic impact of the disease [26].

An international alpha-mannosidosis patient and caregiver-based survey was undertaken and results from the analysis of patient and caregiver proxy responses have been published in Stepien et al. [27]. This study reported that, over time, patients who had not received specialised treatment experienced a decline in walking ability, pain, self-care and mental health. In contrast, in patients receiving ERT or having undergone HSCT, survey responses indicated that the natural progression of alpha-mannosidosis was slower, leading to a different disease trajectory compared with those receiving supportive care alone. The present study examined the impact of the progressive disease burden on the QoL of caregivers of patients with alpha-mannosidosis, at the time of the survey and 5 years prior. Caregiver-reported results allow an exploration of changes in their physical health, mental health, family and social life, relationships and ability to work or attend education. This caregiver-centred analysis aimed to assess the specific impact on caregivers of providing long-term care for individuals with alpha-mannosidosis.

## Methods

Detailed methodology for this international online survey of patients with alpha-mannosidosis and their caregivers has been described previously in Stepien et al. [27]. A short summary of the methodology is provided here, together with results relating to the caregiver at the time of the survey, and 5 years prior.

### Participants

The UK Society for Mucopolysaccharide Diseases (MPS Society) and Rare Disease Research Partners (RDRP) identified 31 clinicians from specialised centres and six LSD/mucopolysaccharidoses (MPS) patient organisations (POs) who then shared an online link/printed QR code to the survey with eligible patients or caregivers. Participation and responses were anonymous to clinicians, POs and the sponsor. Eligible caregivers were those who cared for patients aged ≥ 10 years with a confirmed diagnosis of alpha-mannosidosis. The main caregiver was defined as the individual with daily responsibility for the patient or for at least three days per week. Caregivers were required to be ≥ 18 years old and provide informed consent. Caregivers of patients of all treatment statuses (UP, those who had received HSCT and those on ERT) were eligible for inclusion in the study.

### Survey design

The online survey was available from November 2022 to February 2023 in 13 languages: Czech, Danish, English, Finnish, French, German, Italian, Lithuanian, Portuguese (Brazil), Romanian, Russian, Spanish and Turkish. The caregiver section of the survey comprised questions on their QoL over time (5 years prior and at the time of the survey) for five domains: physical health, mental health (e.g. anxiety, stress, depression, feelings of isolation, grief, blame), family and social life (e.g. inability to go out, restricted in where to go, family issues), relationships with partners, family or friends (e.g. relationship issues, separation, loss of contact with family or friends, lack of understanding) and ability to work or attend education. Questions included those utilising visual analogue scales (VAS), multiple choice questions and open text questions to provide both quantitative and qualitative data. VAS allow measurement of a characteristic across a continuum of values. For this study, caregivers were asked to score the severity of a variable on a scale of 0 (caring for the person with alpha-mannosidosis has no impact on that variable) to 10 (caring for the person with alpha-mannosidosis has a severe impact on that variable) on a horizontal line. VAS are thus useful when assessing change over time in individuals, allowing an arithmetic mean to be calculated for all participants [28]. The survey was hosted on the online Qualtrics^XM^ platform.

### Analysis

Caregiver-reported data for 5 years prior and at the time of the survey were analysed for the five QoL caregiver domains. Categorical variables were reported as frequencies and continuous variables, such as the VAS scores, with descriptive statistics. Mean changes in VAS scores were calculated as the difference in VAS scores between those reported for 5 years prior and those at the time of the survey. A positive mean change suggested a worsening of the caregiver’s QoL and a negative mean change implied a QoL improvement. Mean changes in VAS scores were considered indicative of trends rather than evidence of differences. At the time of the survey, a minimal clinically important difference (MCID) in QoL VAS scores had not been established in alpha-mannosidosis or other LSDs; however, based on a systematic literature review across therapy areas [29], this study described ≥ 1.0 point changes in VAS scores as ‘improvement/worsening’ and < 1.0 point changes in VAS scores as a ‘slight improvement/decline’.

Data were assessed for caregivers of patients receiving ERT, caregivers of patients who had undergone HSCT and those caring for patients not receiving disease-specific treatment (UP). Results from caregivers of patients on ERT were further analysed taking into account the following characteristics of the patients they cared for: age of patient (paediatric [< 16 years old] vs. adults [≥ 16 years old]; 16 years of age is the cut-off point used in clinic in some countries to transition patients from paediatric to adult centres) and length of time on ERT (< 5 years vs. ≥ 5 years).

There was no stratification of caregivers of HSCT patients or UP, due to the small sample size; analysis for these caregivers was presented descriptively. Severity of disease was not collected and was therefore not considered in the analysis. Quantitative analysis was performed using STATA (StataCorp. 2023. Stata Statistical Software: Release 18); qualitative analysis of free text responses used an inductive thematic approach [30].

### Ethical considerations

RDRP conducted this market research in accordance with the British Healthcare Business Intelligence Association’s (BHBIA) Legal & Ethical Guidelines for Market Research [31] and the European Pharmaceutical Market Research Association (EPHMRA). Approval of a Research Ethics Committee was not required because it falls outside the remit of the Research Governance Framework. The confidentiality of information gathered during the study is protected in accordance with applicable data protection legislation and BHBIA’s Legal and Ethical Guidelines. Informed consent was provided by all participants.

## Results

### Caregiver and patient characteristics

Forty-three caregivers (39 mothers; 4 fathers) of 43 patients with alpha-mannosidosis completed the survey. Responses were received from 16 countries: Australia, Brazil, Czech Republic, Denmark, Finland, France, Germany, Italy, Netherlands, Romania, Russia, Saudi Arabia, Spain, Turkey, United Kingdom and USA.

Sixteen caregivers looked after UP who, at the time of the survey, had a median patient age of 29.2 years. Six caregivers looked after HSCT patients with a median patient age of 13.0 years at the time of the survey. These patients had received an HSCT a median of 10.1 years (range 4.3–22.2) before the survey, at a median age of 3.4 years old (range 1.6–7.8 years). Twenty-one caregivers looked after ERT patients of a median age of 19.9 years at the time of the survey, and patients had been on ERT treatment for a median of 5.8 years (range 0.3–12.5 years). The median age of patients at the start of ERT was 16.1 years (range 5.3–37.5 years). Approximately half of ERT patients (11/21) had not started treatment 5 years before the start of the survey; therefore, comparisons of burden for caregivers of this group over the last 5 years incorporated the impact of starting therapy. Full patient demographics and treatment characteristics are shown in Table 1.

**Table 1:** Demographics and treatment characteristics of patients cared for by participants (*n* = 43)

	ERT					HSCT	UP
	All	Paediatrics < 16 years old	Adults ≥ 16 years old	< 5 years on ERT	≥ 5 years on ERT	All	All
**N**	21	4	17	10	11	6	16
**Sex**							
Males	13	3	10	7	6	3	7
Females	8	1	7	3	5	3	9
**Age at the time of the survey**,** years**							
Median	19.9	12.7	29.3	19.3	26.0	13.0	29.2
Mean ± SD	25.1 ± 10.8	12.8 ± 2.3	28.0 ± 9.9	23.8 ± 11.5	26.3 ± 10.5	15.2 ± 4.9	26.6 ± 7.9
Range	10.3–45.7	10.3–15.7	16.3–45.7	10.3–42.2	13.6–45.7	11.4–24.2	11.7–38.4
**Age symptoms were first noticed**,** years**							
Median	1.5	1.0	1.5	1.8	1.1	0.8	3.0
Mean ± SD	2.7 ± 5.0	1.8 ± 2.3	2.9 ± 5.4	1.9 ± 1.3	3.4 ± 6.8	2.7 ± 4.3	6.3 ± 7.5
Range	0.0–23.5	0.0–5.0	0.0–23.5	0.5–5.0	0.0–23.5	0.0–11.3	0.3–24.1
**Age at diagnosis**,** years**							
Median	5.0	7.0	4.0	6.2	4.5	3.4	10.4
Mean ± SD	8.1 ± 7.9	7.0 ± 2.6	8.3 ± 8.7	8.9 ± 8.4	7.3 ± 7.7	4.1 ± 3.1	11.8 ± 7.2
Range	0.0–27.2	4.5–9.6	0.0–27.2	1.3–27.2	0.0–25.2	1.3–9.8	2.6–24.1
**Age treatment started**,** years**							
Median	16.1	8.4	21.0	17.0	16.0	3.4	
Mean ± SD	19.0 ± 10.3	8.2 ± 2.3	21.6 ± 9.8	21.6 ± 10.5	16.7 ± 10.0	4.2 ± 2.6	
Range	5.3–37.5	5.3–10.8	5.7–37.5	9.0–37.5	5.3–34.8	1.6–7.8	
**Length of treatment**,** years**							
Median	5.8	3.6	10.0	1.7	10.2	10.1	
Mean ± SD	6.1 ± 4.4	4.6 ± 4.4	6.4 ± 4.4	2.1 ± 1.4	9.6 ± 2.6	11.0 ± 6.5	
Range	0.3–12.5	1.0–10.3	0.3–12.5	0.3–4.7	5.8–12.5	4.3–22.2	

UP were defined as individuals who were not receiving disease-specific treatment

ERT=enzyme replacement therapy; HSCT=haematopoietic stem cell transplantation; SD=standard deviation; UP=untreated patients

### Caregiver physical health

#### Overall changes

**Fig. 1:**
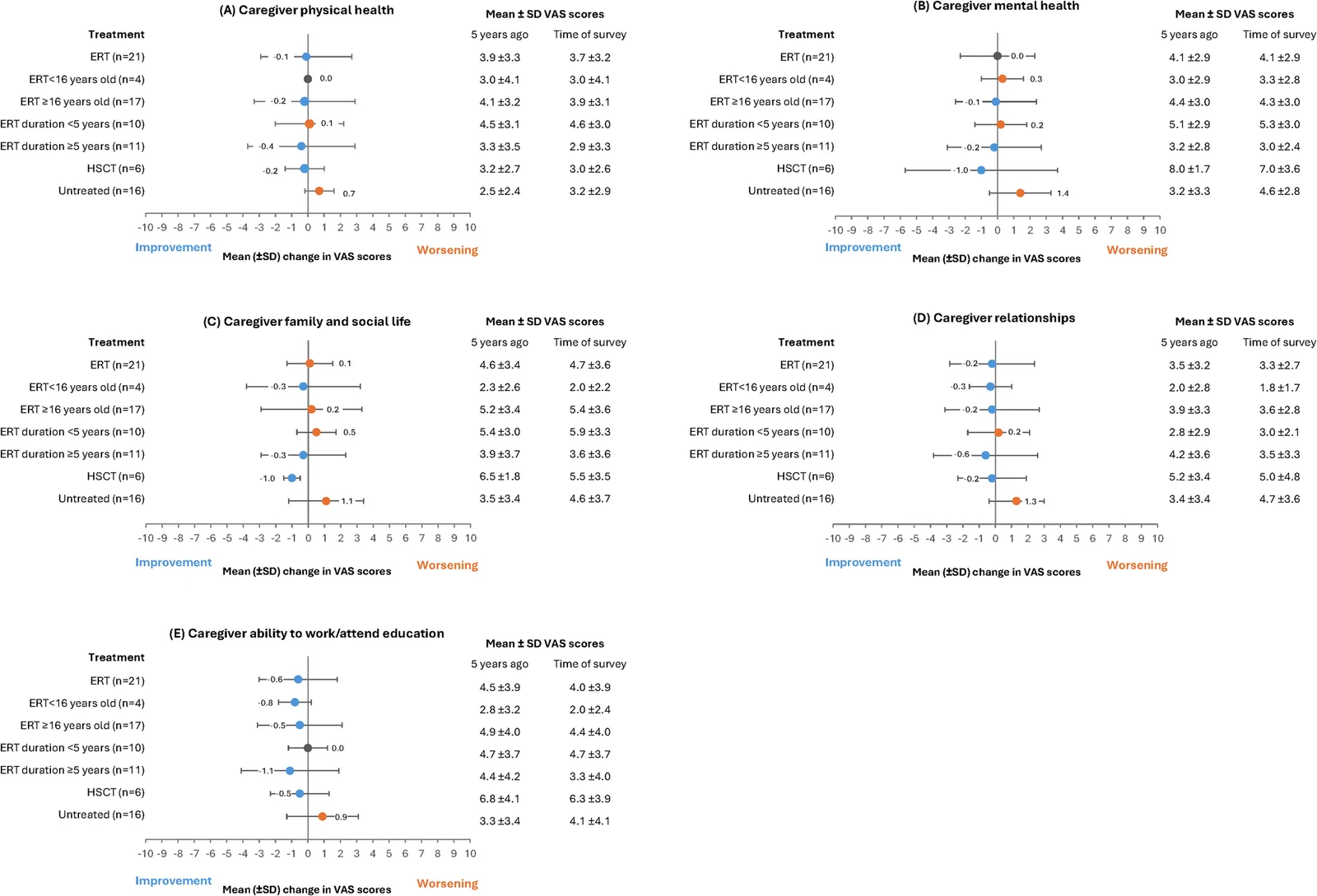
Mean ± SD changes in VAS scores over 5 years per treatment group for caregivers’ (**A**) physical health; (**B**) mental health; (**C**) family and social life; (**D**) relationship with partners, family or friends; (**E**) ability to work/attend education. Blue datapoints indicate improvements/slight improvement, orange datapoints indicate declines/slight declines and grey datapoints indicate no change in mean VAS score. ERT=enzyme replacement therapy; HSCT=hematopoietic stem cell transplantation; SD=standard deviation; VAS=visual analogue scale

Five years prior to survey completion, mean ± standard deviation (SD) VAS physical health scores were worst for caregivers of ERT patients (3.9 ± 3.3), followed by those caring for HSCT patients (3.2 ± 2.7) and UP (2.5 ± 2.4). Over the course of 5 years, physical health slightly improved in caregivers of ERT and HSCT patients (mean changes in VAS scores of − 0.1 ± 2.8 and − 0.2 ± 1.2, respectively) and slightly declined for caregivers of UP (+ 0.7 ± 0.9) (Fig. 1A; Additional Table 1).

Over time, mean VAS scores slightly improved for caregivers of patients on ERT who at the time of the survey were adults (-0.2 ± 3.1), started ERT as adults (-0.5 ± 3.6) or had been on ERT for ≥ 5 years (-0.4 ± 3.3), while change in mean physical health scores remained stable for caregivers of paediatric patients. Physical health slightly declined for caregivers of those who started ERT at paediatric age (+ 0.3 ± 0.7) or were on ERT for < 5 years (+ 0.1 ± 2.1) (Fig. 1A; Additional Table 1).

#### Individual changes

Among caregivers of patients receiving ERT, physical health remained stable for 11/21 (52.4%), worsened for 7/21 (33.3%) and improved for 3/21 (14.3%) over the 5-year period. Equal numbers of caregivers of HSCT patients reported improvements, stabilisation and worsening in physical health (for all, 2/6 [33.3%]). For caregivers of UP, 9/16 (56.3%) respondents reported no change in physical health whilst 7/16 (43.8%) experienced worsening over the 5 years. Further details at individual level are described in Additional Fig. 1.

Caregivers expressed concerns regarding the physical demands of caring for their child with alpha-mannosidosis, particularly as the disease progressed and as they aged themselves (Table 2, **row 1**).

**Table 2:** Selection of caregivers’ quotes: insights about the caregiver burden of caring for a patient with alpha-mannosidosis

**Physical health**	
1	**Physical demands as disease and carer’s age progress:** “As his stability deteriorated, he needed more and more help with all normal tasks during the day. He became less and less independent and could not meet with people of his own age when he wanted to but needed our help. The level of assistance required constantly increased. He is now entirely dependent on us […] Since my husband and I are both 64 years old, it’s starting to be difficult for us”** Caregiver of an adult UP.** “The need for help is increasing. The burden is the wheelchair, immobility. We’re getting older and care is more demanding. Services are inadequate.”** Caregiver of an adult UP**.
**Mental health**	
2	**Worry about the unknown extent of the patient’s discomfort and pain:** “[Paraphrased] I am not sure how much pain [child’s name] was experiencing when she was younger as she couldn’t express herself properly and she could not understand what we were asking from her, until she turned 4/5 years old. She used to cry quite frequently, and we would feel saddened as we saw she was in pain but we weren’t sure how to help her […]” Caregiver of a paediatric patient who received HSCT. “I am not able to ascertain general pain or discomfort due to limited communication ability, but it concerns me greatly” **Caregiver of a paediatric patient who received HSCT.**
3	**Worry as patients become more independent:** “She lives in one of her employer’s supervised facilities but is with us at the weekend and for treatment. We are very limited by her limitations as we are always afraid that she will fall and sustain a fracture.” **Caregiver of an adult patient on ERT.**
4	**Guilt:** “For us parents it was difficult to give her up to a [care]home – for a very long time we had very guilty consciences, […].” **Caregiver of an adult patient on ERT.**
**Family and social life**	
5	**Active social life and meeting other families:** “We’ve never had a social life problem. We’ve been out a lot, we’re going to a football game, we’ve even been on the field several times with the players of the team we cheer for. We go to the movies, park, church, everywhere” **Caregiver of an adult UP**. “Now there is a better quality of life because we are now involved with groups that support disability. Got to know lots of people with differing issues relating to disability and mental health. There is more socialising with different groups, going out for coffee, cinema etc. […] now there is more opportunity to mix with groups of people with similar issues.” **Caregiver of an adult UP.**
6	**Significant impact on social life and holidays:** “We often think about the chances of her being in pain when organising daily activities. This greatly limits us on what activities we can do for the day, who we can encounter and what type of family vacations we are going to have.” Caregiver of a paediatric patient who received HSCT. “For us parents it was a difficult time, as two people were always needed to help with going to the toilet. As her mother, I was also able to do that alone to some extent, but not my husband and so I was also trapped in the house the whole year.” Caregiver of an adult ERT patient. “He was pretty much independent up until the age of roughly 15 […]. As his stability deteriorated, he needed more and more help with all normal tasks during the day. […] He is now entirely dependent on us. […]. There is no facility within 100 km of where we live, where I could leave [child’s name] for a few days and go off on holiday […] it’s starting to be difficult for us. It really limits our social activities.” **Caregiver of an adult UP.**
7	**Difficulties adapting to new and unfamiliar environments:** “A huge problem we are experiencing is increased confusion and anxiety. It’s difficult to do any new activities as he is dependent on environments which are familiar to him. He will not be able to manage new places and new holidays are impossible. […] Full support was always required. Moreover, now he has got bigger, personal care needs and access make going out more difficult.” **Caregiver of an adult UP.**
**Relationship with partners**,** family or friends**	
8	**Travel-related limitations:** “Her family life is very supportive. She lives with both of her parents and her father handles all her care. She has aunts and uncles that live within driving distance […]. However, traveling to see family who do not live in our city has become difficult due to her need for supportive equipment.” **Caregiver of an adult UP.**
9	**Strain in caregiver-patient relationship:** “The development of the disease has increased the burden on the family. My husband is 66 years old and he finds it difficult to deal with the fact that our daughter is often unfriendly to us.” **Caregiver of an adult ERT patient.**
**Ability to work or attend education**	
10	**Care responsibilities shaping work and study opportunities:** “I am a full-time carer, and I am not doing other things alongside it, I am not working nor studying.” Caregiver of a paediatric HSCT patient. “While my husband was studying, I was with the children, now he is with them, I am studying. The special school has its own work schedule, which does not coincide with the general one. Difficult but we manage.” **Caregiver of an adult ERT patient.**

UP were defined as individuals who were not receiving disease-specific treatment

ERT=enzyme replacement therapy; HSCT=haematopoietic stem cell transplantation; UP=untreated patients

### Caregiver mental health

#### Overall changes

Mean ± SD VAS mental health score for 5 years prior was worst for caregivers of HSCT patients (8.0 ± 1.7), followed by those caring for ERT patients (4.1 ± 2.9) and UP (3.2 ± 3.3). Over time, there was an improvement in mental health scores for caregivers of HSCT patients (mean change in VAS score: -1.0 ± 4.7). Mental health scores remained stable for caregivers of ERT patients (0.0 ± 2.3). Although there was a worsening in mental health for caregivers of UP (+ 1.4 ± 1.9), their mean VAS scores were better 5 years ago and at the time of survey participation compared with caregivers of HSCT and ERT patients (Fig. 1B; Additional Table 1).

Over 5 years, mean mental health VAS scores showed a slight improvement for caregivers of adult ERT patients (-0.1 ± 2.5), of those starting ERT as adults (-0.6 ± 2.7) and those who had received ERT for ≥ 5 years (-0.2 ± 2.9). Although mean scores slightly declined for caregivers of paediatric ERT patients (+ 0.3 ± 1.3), the mean score for these caregivers was higher than those caring for adult ERT patients, both 5 years ago and at the time of the survey. Scores indicated a slight decline in mental health for the caregivers of patients who started ERT at paediatric age (+ 0.8 ± 1.5) and of those receiving ERT for < 5 years (+ 0.2 ± 1.6) (Fig. 1B; Additional Table 1).

#### Individual changes

Mental health for caregivers of ERT patients remained stable in 11/21 (52.4%), worsened in 6/21 (28.6%) and improved in 4/21 (19.0%) (Additional Fig. 2). Equal numbers of caregivers of HSCT patients reported improvements, stabilisation and worsening in mental health (for all, 2/6, 33.3%). Mental health worsened in 10/16 (62.5%), stabilised in 4/16 (25.0%) and improved in 2/16 (12.5%) of caregivers of UP. Further details at individual level are described in Additional Fig. 2.

**Fig. 2:**
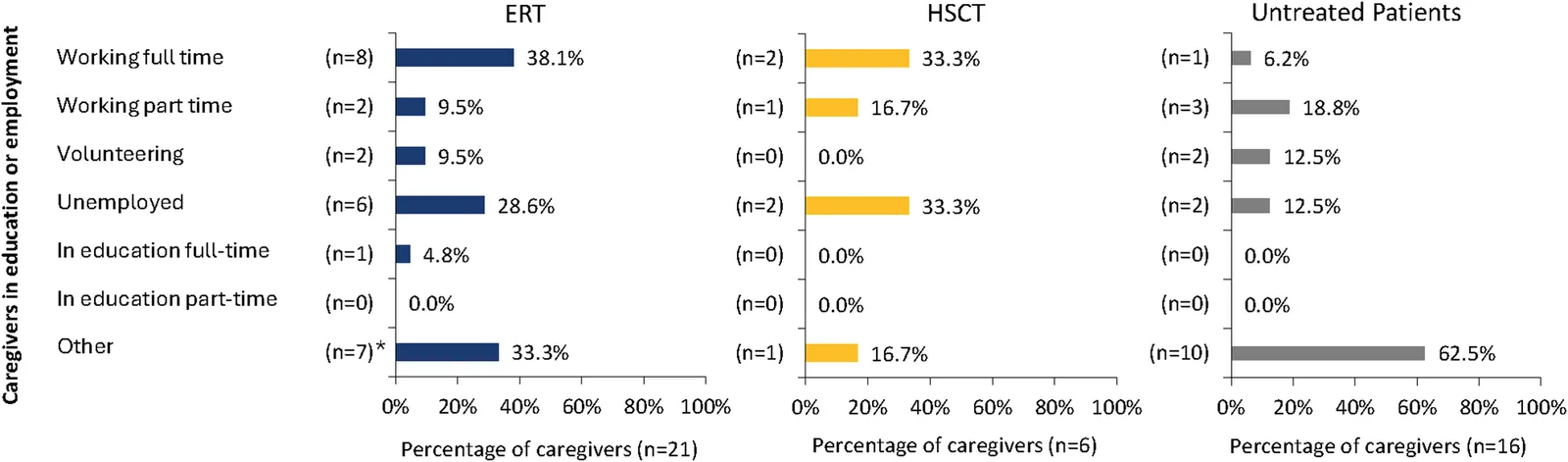
Status of caregiver employment or education at the time of the survey. Caregivers could select more than one response, if applicable. *Among the caregivers who responded “other”, three ERT caregivers and five UP caregivers reported being retired in the associated free-text response. ERT = enzyme replacement therapy; HSCT = haematopoietic stem cell transplantation

Caregivers described how upsetting it was to see their child being in pain and not being able to help, along with their worry over the unknown extent of their discomfort because of the child’s limited communication abilities or young age (Table 2, **row 2**). Although some adult patients were able to become more independent, the survey showed that caregivers were constantly worried about their child (Table 2, **row 3**). Parents also reported experiencing feelings of guilt associated with placing their child into a care home (Table 2, **row 4**). In addition, caregivers reported that dealing with schooling and employment processes on behalf of the patient can be exhausting and time-consuming for the whole family.

### Caregiver family and social life

#### Overall changes

Reported burden on family and social life 5 years prior to the survey was worst for those caring for HSCT patients (mean VAS score, 6.5 ± 1.8), followed by caregivers of ERT patients (4.6 ± 3.4) and then caregivers of UP (3.5 ± 3.4). Over the 5-year period there was an improvement in scores for caregivers of HSCT patients (mean change in VAS score: -1.0 ± 3.5), a worsening for caregivers of UP (+ 1.1 ± 1.4) and a slight decline for caregivers of ERT patients (+ 0.1 ± 2.3) (Fig. 1C; Additional Table 1).

There was a slight improvement in family and social life over the 5 years for caregivers of paediatric ERT patients (-0.3 ± 0.5) and those caring for patients on ERT for ≥ 5 years (-0.3 ± 3.1). There was a slight decline for those caring for: adult ERT patients (+ 0.2 ± 2.6), patients who started ERT at < 16 years (+ 0.2 ± 0.8) and those on ERT for < 5 years (+ 0.5 ± 1.2) (Fig. 1C; Additional Table 1).

#### Individual changes

For caregivers of ERT patients, family and social life was stable in 10/21 (47.6%), worsened in 8/21 (38.1%) and improved over time for 3/21 (14.3%). Half of caregivers of HSCT patients (3/6, 50%) experienced an improvement in family and social life, 1/6 (16.7%) a stabilisation and 2/6 (33.3%) a worsening. Most caregivers of UP (9/16, 56.3%) reported a worsening in family and social life, while 6/16 (37.5%) and 1/16 (6.3%) reported stabilisation and improvement, respectively. Further details at individual level are described in Additional Fig. 3.

Two caregivers of UP mentioned that they had never had a problem with their social life and continued to enjoy multiple social activities, especially when accessing support groups where they were able to meet other families with similar challenges (Table 2, **row 5**).

In contrast, most caregivers described a significant negative impact of caring for the patient on their ability to go out, to participate in normal activities and to go on family holidays (Table 2, **row 6**). The challenges of taking part in activities or holidays in new environments were also highlighted, especially as the disease progressed (Table 2, **row 7**).

### Relationship with partners, family or friends

#### Overall changes

The impact of the disease on caregivers’ relationships 5 years prior was similar for those caring for patients treated with ERT and UP (mean VAS scores: 3.5 ± 3.2 and 3.4 ± 3.4 respectively) but was larger for caregivers of HSCT patients (5.2 ± 3.4). Over the 5-year period, relationships slightly improved for caregivers of ERT and HSCT patients (mean change in VAS score: -0.2 ± 2.6 and − 0.2 ± 2.1, respectively) but worsened for caregivers of UP (+ 1.3 ± 1.7) (Fig. 1D; Additional Table 1).

Mean ± SD VAS scores for caregivers’ ratings of their relationships slightly improved over the 5 years for caregivers of both paediatric and adult ERT patients (-0.3 ± 1.3 and − 0.2 ± 2.9, respectively), of those who started ERT at paediatric and adult age (-0.1 ± 1.3 and − 0.3 ± 3.4, respectively) and of patients on ERT for ≥ 5 years (-0.6 ± 3.2). Relationship scores slightly declined over the study period for caregivers of patients on ERT for < 5 years (+ 0.2 ± 1.9) (Fig. 1D; Additional Table 1). 

#### Individual changes

For caregivers of patients on ERT, relationship scores remained the same for 12/21 (57.1%), worsened for 5/21 (23.8%) and improved for 4/21 (19.0%) over the 5 years. Of the six caregivers of patients treated with HSCT, two reported a worsening in relationships, two a stabilisation and two an improvement. Relationship scores worsened for 9/16 (56.3%) of caregivers of UP, stabilised for 6/16 (37.5%) and improved for 1/16 (6.3%). Further details at individual level are described in Additional Fig. 4.

Positive and supportive family relationships were commonly mentioned by caregivers; however, difficulties in travelling to see family based far away were mentioned (Table 2, **row 8**) and, in at least one case, the family had relocated to a different country to better support their child with the disease, leaving other family and friends behind. Limitations on outings and social activities noted by many caregivers would also be expected to have significant negative impacts on social interactions and relationships. In addition, strains in the relationship between a caregiver and the patient can unfortunately occur, as reported by one caregiver (Table 2, **row 9**).

### Ability to work or attend education

#### Overall changes

Five years before the survey was carried out, the mean VAS score describing the ability of caregivers to work or attend education was worst for caregivers of HSCT patients (6.8 ± 4.1), followed by those caring for ERT patients (4.5 ± 3.9) and UP (3.3 ± 3.4). Over the 5-year period, there were slight improvements for caregivers of ERT and HSCT patients (mean change in VAS score: -0.6 ± 2.4 and − 0.5 ± 1.8, respectively) and a slight decline for those caring for UP (+ 0.9 ± 2.2) (Fig. 1E; Additional Table 1).

There was an improvement in the ability to work or attend education for caregivers of patients on ERT for ≥ 5 years (mean change in VAS score: -1.1 ± 3.0). Slight improvements were observed for caregivers of both paediatric and adult ERT patients (-0.8 ± 1.0 and − 0.5 ± 2.6, respectively) and caregivers of patients starting ERT at both paediatric or adult age (-0.2 ± 1.5 and − 0.8 ± 2.9, respectively). The ability to attend work or education remained stable for those caring for patients on ERT for < 5 years (Fig. 1E; Additional Table 1).

#### Individual changes

At the time of the survey, 10/21 (47.6%) caregivers of ERT patients and 3/6 (50.0%) caregivers of HSCT patients were working (full-time or part-time), compared with 4/16 (25.0%) caregivers of UP who were in employment. Of the caregivers of ERT patients working full-time, 6/8 cared for adult ERT patients and 5/8 cared for patients on ERT for ≥ 5 years. Among six caregivers of ERT patients who were unemployed, four cared for adult ERT patients. Of the 18 caregivers who responded ‘other’ for their employment/education status, 8 (three ERT caregivers, five UP caregivers) reported to be retired in the associated free-text response (Fig. 2). Further details at individual level are described in Additional Fig. 5.

One caregiver highlighted the need to work to pay for expensive supportive care for their child at a specialised facility, whilst other caregivers described their role of carer as full-time, and the need to alternate with their partner for working or pursuing education (Table 2, **row 10**).

## Discussion

This is the first international study of the QoL burden experienced by caregivers of patients with alpha-mannosidosis, and how this burden may change over time for caregivers of patients receiving disease-modifying treatment or supportive care only. The survey revealed that from five years prior to the time of survey participation, caregivers of patients who had received HSCT had improvements or slight improvements in all five of the QoL domains included in the survey, while caregivers of patients on ERT experienced stable or slightly improved scores in four of the five domains, with those who were on ERT for ≥ 5 years generally experiencing more improvements than those who were on ERT for < 5 years. In contrast, caregivers of UP experienced worsening or slight declines in all five QoL domains. Results from the patients cared for by these caregivers reported in Stepien et al. [27] mirror these caregivers’ results: treated patients participating in the survey showed a slower natural progression of the disease compared to UP. It should be noted that HSCT patients were considerably younger than ERT patients and UP, with UP being the oldest. The limitations of this descriptive analysis prevent assessment of a causal relationship between treatment status and caregiver QoL. In particular, small participant numbers in some subgroups and differences in patient age across the HSCT patients, ERT patients and UP may explain some of the individual differences in VAS scores observed in this study, which are discussed in turn, below.

### Caregivers of HSCT patients

Overall, caregivers of HSCT patients experienced an improvement in residual burden over the 5-year study period, with the largest improvement in mental health and social life. This may be due to improvements in patient self-care and a less pronounced decline in walking ability among the HSCT patients, which were revealed by patient data from the same survey [27]. Such improvements were likely to have contributed to better caregiver QoL, with caregiver testimonies in the current study revealing that patient mobility has an important impact on caregivers.

It should however also be noted that caregivers of HSCT patients reported some of the highest (worst) VAS scores, both 5 years prior to and at the time of survey participation, across 4/5 of the QoL domains (mental health, family and social life, relationships and work/education). This was despite most patients having received HSCT a median of 10.1 years prior to the survey. All the patients who received HSCT in the current study were children (age range 1.6–7.8 years) at the time of the procedure and were notably younger than the ERT patients, indicating treatment intervention at a much younger age compared with ERT. The anxiety of caregivers over the outcome of HSCT and whether their young child would achieve developmental milestones in the years after treatment may therefore have negatively impacted multiple QoL domains, although all parents of children with alpha-mannosidosis would likely feel similar levels of anxiety about their child’s development, regardless of which treatment they were receiving. In addition to the younger age of HSCT versus ERT and UP, poorer QoL among caregivers of HSCT patients could also have been a result of the burden of dealing with the impacts of the procedure, the challenges associated with hospital appointments and assessments (including family separation and balancing hospital visits with home/family life), worry and stress about risks (including potential relapse and mortality), perhaps unmet expectations and the number of years post-treatment [32, 33]. This is supported by several reviews of caregiver burden before, during and after HSCT, which have described significant negative impacts on caregivers across the HSCT trajectory and on multiple domains, including mental wellbeing, QoL and relationships [34–36]. In a study involving caregivers of patients with metachromatic leukodystrophy (MLD) from six countries (including USA and Europe) who received a transplant, caregivers reported being forced to relocate from their homes, miss work and make arrangements for their other children around the time of the transplant [18].

### Caregivers of ERT patients

Caregivers of ERT patients appeared to experience slight improvements in physical health, relationships and ability to work/attend education, but no change in mental health and a slight decline in family/social life. Symptom benefits of ERT include improved endurance and pulmonary function, which might reduce patient dependence on caregivers [11–13].

### Caregivers of patients on ERT for < 5 versus ≥ 5 years

Over the 5-year study period, caregivers appeared to improve slightly in all QoL domains when patients had been on ERT for ≥ 5 years, with a more notable improvement in their ability to work or attend education. This could be due to slight improvements in self-care, pain/discomfort and mental health over the study period amongst the patients on ERT ≥ 5 years [27]. The lack of improvement for caregivers of patients who started ERT during the 5-year period of this study may reflect the burden of adjusting to the management and logistics required for receiving weekly treatment infusions and the perceived uncertainty of the outcomes in the short term.

#### Age of ERT initiation

Slight improvements in mental health were seen among caregivers of patients who started ERT as an adult. These improvements may be a result of coming to terms with and adapting to life with the disease, having accessed support organisations and, most likely, relief and hope at the introduction of ERT after years with no available treatments. The caregiver mental health results mirror the large numerical improvements in mental health VAS scores reported by patients who started ERT as adults, previously reported in our patient data, versus the decline in patients who started ERT at paediatric age [27]. Conversely, a slight decline in mental health over the 5-year study period was reported for caregivers of patients who started ERT as children. This was possibly due to the need to process a relatively recent diagnosis along with a wealth of new information, juggling care of other siblings, challenges of accessing treatment and stress caused if their child presented with pain and had difficulty in communicating the level, type and location of pain. It has also been suggested that an elevated caregiver burden, based on the caregiver’s perception of the child’s disease progression, could be a risk factor negatively impacting their child’s QoL [37].

#### Age of ERT patients at the time of the survey

Caregivers of ERT patients who were adults at the time of the survey appeared to experience a slight decline in family and social life scores over the 5-year study period. This decline could reflect the sustained or increasing care demands of patients who remain dependent on their caregivers as they transition into adulthood, a period during which greater independence would ordinarily be expected. Moreover, patients who did not initiate ERT until adulthood were likely to already present with ataxia, markedly reduced mobility, or wheelchair dependence [3, 4], which could impose increased limitations on family/social life for the whole family, affecting the planning of daily activities and holidays. The requirement for weekly infusions and the associated lifestyle adjustments may have further contributed to the slight decline in caregiver family and social life VAS scores. These factors also increase the day-to-day physical demands on caregivers, which become particularly challenging as caregivers themselves age.

For those who cared for ERT patients who were children at the time of the survey, there was only a slight decline in mental health scores. As has already been discussed with caregivers of children who had received HSCT, caregiver mental health may have been negatively impacted by the anxiety and complex care needs associated with caring for a young child with alpha-mannosidosis.

### Caregivers of UP

Caregiver QoL burden over the 5 years of the study seemed to increase across all domains for carers of UP, reflecting the expected natural disease progression of alpha-mannosidosis in patients. A decline in caregivers’ QoL has been attributed to the substantial physical and emotional demands of patient care, with anxiety and depression particularly pronounced in those caring for patients experiencing rapid disease progression or cognitive impairment [38]. In this study, caregivers of UP exhibited the largest numerical decline in mental health VAS scores, potentially reflecting the cumulative burden of dealing and managing patient deterioration, as well as addressing the patients’ own mental health, which was reported in Stepien et al. to be most severe over this five-year period for UP [27]. This study did not collect the age of caregivers; therefore, it cannot be inferred if caregivers of UP were older and/or had health issues of their own. However, UP were older than the HSCT and ERT patients, and five caregivers of UP reported to be retired, which may be indicative of older age.

Although the caregivers of UP had worsening mental health over time, their mean mental health VAS scores were generally better than those caring for treated patients, both 5 years prior and at the time of survey completion. This might be attributed to the older age of UP versus treated patients at the time of survey participation. Quality of life was likely to have been higher amongst the caregivers of older patients, due to greater patient independence compared with young children and a longer time to adapt following diagnosis. Together with higher rates of caregiver retirement amongst UP caregivers, this may partly explain the comparatively lower mental health burden reported by caregivers of UP at both time points.

### Across-group observations

High levels of unemployment across all caregiver groups highlighted the demands and burden of caring for patients with alpha-mannosidosis. A previous study reported that nine caregivers of patients with alpha-mannosidosis spent a mean of 16.6 hours per day providing care, with 78% unable to work full time [22]. The time required to care for patients and travel to and attend frequent appointments in multiple locations can make it extremely difficult for carers to consider full-time employment. At the time of our survey, approximately half of the caregivers of treated patients (HSCT or ERT) were pursuing education or were employed either full-time or part-time, compared with just a quarter of caregivers of UP. The financial implications of this situation might have increased the levels of anxiety amongst caregivers.

The testimonies and descriptions received from caregivers in the open text questions of the survey are very informative regarding the burden and challenges that they face. The level of assistance and care required by the patients with alpha-mannosidosis included in this study increased over time. Older carers expressed their concerns regarding the future care of the patient and were more likely to find the physical demands of caring for the patient (and the patient’s behavioural issues) more challenging. Caregivers experienced upset, stress and significant impacts on their ability to perform daily activities, maintain a social life and go on holidays; the need to work was highlighted but the rates of employment were low. Feelings of guilt were also described by caregivers, in particular if they must move the patient to a care home. Reports in the current study are in agreement with and extend the findings from the UK survey of nine caregivers of patients with alpha-mannosidosis, who also described feelings of stress and anxiety, pressure on relationships and negative impacts on social lives and employment [22]. In addition, these significant impacts on mental health, daily life, relationships, social functioning and employment are similar to those described by caregivers of patients with other LSDs such as MLD, MPS III (Sanfilippo syndrome type B and Sanfilippo A syndrome) and MPS type II (Hunter syndrome) [17, 18, 23–25, 35]. Although the results of this study should be interpreted with caution and no causal relationship between treatment status and caregiver QoL can be assumed without further larger-scale investigation, they suggest that the benefits of early intervention with disease-modifying therapy could potentially extend to caregiver QoL. The benefits of early intervention with HSCT or ERT on delaying disease progression are well established [11, 13, 14, 39, 40] and were supported by the patient results from this survey [27]. Caregivers of patients who had initiated ERT treatment ≥ 5 years earlier reported slight improvements in all QoL domains, potentially reflecting both the cumulative benefits of treatment and the advantages of earlier intervention, as well as the caregivers’ adaptation to the ongoing demands of therapy. Furthermore, the declines in QoL observed for UP caregivers imply that the burden associated with the natural progression of alpha-mannosidosis outweighs the combined logistical and treatment burden of HSCT and ERT. Strengths and limitations of the study have been previously discussed in Stepien et al. [27]. The survey included a relatively large international sample of caregivers for an ultra-rare disease. However, disease severity is likely to affect caregiver burden; this was not captured by the study, although differences in the median age that symptoms were first noticed were recorded and were relatively small within groups. Systematic reviews across other therapeutic areas have identified patient disease severity, and therefore functional dependency and symptom burden, as key factors influencing caregiver outcomes [41, 42]. Caregivers of patients with more advanced disease consistently report a greater caregiving burden and reduced QoL compared with caregivers of patients with less severe disease.

Differences in the ages of patients and caregivers were not taken into account; caring for an adult with an untreated severe progressive disease is inevitably very different to caring for a child who is undergoing treatment, especially when the carer is of an older age themselves. As described earlier, HSCT patients were predominantly of paediatric age, whilst UP were predominantly adults; a causal relationship between disease-modifying treatment and caregiver burden therefore cannot be assumed. Caregiver demographics other than parental relationship with the patient were beyond the scope of the study and were not recorded. The use of VAS for assessing changes over time has been previously described as providing simplicity, reliability, validity and practicality and the ability to capture additional aspects of QoL not included in other instruments or questionnaires [43]. However, VAS tools cannot robustly identify the specific determinants impacting a caregiver’s QoL rating [44]. Therefore, VAS changes from 5 years prior to the time of survey participation in this study likely reflect a combination of natural history progression, treatment initiation effects and logistical/administration treatment burden, rather than treatment effect alone. Self-assessment of caregiver burden can also be impacted by differences in language and cultural expectations around caregiving [45]. Although professional translators were utilised to translate the VAS into local language, linguistic and cultural validation of the VAS tool was not performed across different participating countries in this study. In the absence of an established MCID for QoL VAS scores at the time of the study, pre-defined VAS cut-off values were not implemented.

Although the sample size of 43 caregivers restricted the ability to perform statistically robust subgroup analyses and precluded any statistical conclusions, it allowed for the identification of descriptive trends. Caregiver numbers in some of the subgroups were particularly small. For example, only six caregivers of HSCT patients and four caregivers of paediatric ERT patients took part in the study; they were not excluded from the analysis since any data in this patient/caregiver group is valuable for understanding caregiver experience. The survey did not ascertain whether the working status of parents reflected employment limitations related to their child’s condition; therefore, non-working status may have included individuals who were retired or not employed for reasons unrelated to the disease. Recall bias of caregivers remembering past events 5 years prior could lead to overestimation or underestimation of experiences, but this bias was equally applicable across responses, giving no advantage to any of the treatment groups. Despite these limitations, our qualitative analysis provided valuable insights into the burden experienced by those caring for patients with alpha-mannosidosis and raised some important considerations for future, larger-scale studies in which subgroup analyses might be feasible. For instance, a larger caregiver-focused study would allow for the collection of additional objective measures of caregiver burden (such as travel requirements, income loss, individual healthcare costs and caregiving hours) and for the investigation of potential differences in healthcare access, socioeconomic status or VAS response style between participants. Furthermore, comparator cohorts of caregivers in other lysosomal storage diseases, rare neurodevelopmental disorders or chronic neurological conditions could be included. Such comparisons would help contextualise the magnitude and nature of caregiver burden observed in alpha-mannosidosis, whilst strengthening construct validity and providing insights on which aspects of caregiver burden are disease-specific versus common across chronic rare diseases. It would also be interesting to explore how between-country differences in national income status and health and social care systems could impact the degree of burden experienced by caregivers, owing to potential differences in available support such as wage protection, health and social insurance or benefits, public services and local infrastructure [46, 47]. No respondents from low-income countries took part in the current study, likely reflecting the barriers to participating in medical research that these individuals face [48]. Another factor for future investigation is the prevalence of non-communicable disease (NCD) in a given geographic area, potentially impacting not only the caregiver’s own health, but also the quality of healthcare and social care services available to caregivers, particularly in lower-middle-income or low-income countries with high NCD burden [49–51].

## Conclusion

This study provides the first international insight into the longitudinal QoL burden experienced by caregivers of patients with alpha-mannosidosis, exploring how this burden may evolve over time in relation to treatment status. Overall, caregivers faced substantial challenges, with impacts on mental health, social relationships, daily activities and employment, reflecting the profound and sustained demands of caring for someone with a rare, progressive disease. Caregivers of untreated patients generally experienced a progressive worsening of burden across all QoL domains, whereas those caring for patients treated with HSCT or receiving ERT generally reported stabilisation or improvement, particularly when treatment was initiated early and sustained. Although a causal relationship between disease-modifying treatment and caregiver QoL cannot be assumed based on limitations of the analysis, combined with previous findings [37], these results suggest that timely, disease-modifying interventions may indirectly benefit caregivers by slowing disease progression and reducing care demands. Given the limited research on caregiver experiences in alpha-mannosidosis, these results highlight the importance of integrating caregiver perspectives into clinical decision-making, developing targeted support strategies and informing rare disease policy and resource allocation. Recognising and addressing the social, emotional and practical impact on families will be critical for improving outcomes and supporting the long-term wellbeing of both caregivers and patients living with alpha-mannosidosis.

## Supplementary Information

Below is the link to the electronic supplementary material.

Supplementary Material 1: Additional Table 1. Mean VAS scores now and 5 years ago and mean change in VAS scores for caregivers’ physical health, mental health, family and social life, relationships and ability to work or attend education. Additional Figure 1. Caregivers’ individual changes in physical health. Additional Figure 2. Caregivers’ individual changes in mental health. Additional Figure 3. Caregivers’ individual changes in family and social life. Additional Figure 4. Caregivers’ individual changes in their relationship with partners, family or friends. Additional Figure 5. Caregivers’ individual changes in their ability to work or attend education

Supplementary Material 2: Additional file 1: Plain language summary of manuscript

## Data Availability

The raw dataset is not publicly available to ensure the protection of patient confidentiality within the small population of alpha-mannosidosis patients and caregivers.

## Abbreviations

BHBIA: British Healthcare Business Intelligence Association
EPHMRA: European Pharmaceutical Market Research Association
ERT: Enzyme replacement therapy
HSCT: Haemopoietic stem cell transplantation (allogenic)
LSD: Lysosomal storage disorder
MLD: Metachromatic leukodystrophy
MPS: Mucopolysaccharidoses
POs: Patient organisations
QoL: Quality of life
SD: Standard deviation
UP: Untreated patients
USA: United States of America
VAS: Visual analogue scales
