# Supplementary material for: Quality of life and burden of caregivers of patients with alpha-mannosidosis: an international survey
Source: Orphanet J Rare Dis. 2026 Jul 22;21:385. doi: 10.1186/s13023-026-04496-y (PMC13732844; doi:10.1186/s13023-026-04496-y)
Supplement: Supplementary file 1 [file 13023_2026_4496_MOESM2_ESM.pdf]

Quality of life and burden for caregivers of patients with alpha-mannosidosis: an international survey

Supplemental information to Hennermann et al. *Orphanet Journal of Rare Diseases*. 2026.

Authors: JB Hennermann, N Guffon, S Thomas, KM Stepien, C Lampe, NM Muschol, MJ Ballesta-Martínez, J Cruz, M López-Rodríguez, A Barth, AM Lund, V Plaiasu, C Donati, HM Morgan, A Ballabeni, M Magner.

This is a plain language summary of an article about the evolution of quality of life experienced by caregivers of patients with alpha-mannosidosis, which was published in *Orphanet Journal of Rare Diseases* in 2026.

What is alpha-mannosidosis (AM)?

AM is an extremely rare, inherited condition in which the enzyme called alpha-mannosidase does not work properly or is missing.

Alpha-mannosidase is responsible for helping clear waste from the cells in our body. Without this enzyme, **oligosaccharides** (complex nutrients that the body uses for several different functions) build up in the cells, which can make it harder for them to function properly.

People living with AM can experience a variety of symptoms that affect different organs, and these can become more severe over time.

Why was the study done?

As an ultra-rare disease, more and more is understood about how AM affects people from a medical perspective, but less is known about how AM impacts people living with AM, and those who care for them, in the long-term and in their day-to-day life. To learn more, an international survey was sent to people with AM and their caregivers. The results of this survey were used for a study on how the disease impacts the lives of people with AM, which was published in 2025.

The goal of this study was to better understand how AM impacts day-to-day quality of life for **caregivers** over time, by comparing their experiences from five years ago to the time of the survey.

What type of questions were included in the survey?

The people who took part in the survey answered both multiple-choice and open-ended questions. They also rated different aspects of life for a caregiver of a person with AM, using a **Visual Analog Scale (VAS)**. This included rating how good or bad they perceived their physical health, mental health, family and social life, relationships, and ability to work or attend education to be.

What is a VAS?

VAS is a measurement tool where participants mark the intensity of a feeling from none to maximum.

Caregiving has no impact at all on my physical health										Caregiving has severe impacts on my physical health
0	1	2	3	4	5	6	7	8	9	10

Now

5 years ago

Example VAS

Participants were asked to describe their situation now and to recall how things were five years ago. Comparing these answers helped show which areas have worsened, stayed the same, or improved.

What treatments are available for AM?

There are few treatments for AM, with some patients only receiving supportive care and management of symptoms as they appear.
The two main options are:

1. **Hematopoietic stem cell transplant (HSCT):** replaces diseased bone marrow with healthy cells that produce the missing enzyme.
2. **Enzyme replacement therapy (ERT):** replaces or supplements the missing or impaired enzyme.

Who took part in the survey?

43 caregivers from **16 countries** took the survey:

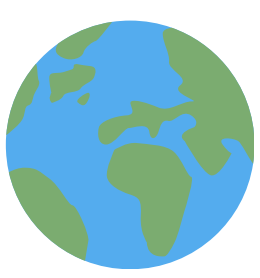

- Australia
- Brazil
- Czech Republic
- Denmark
- Finland
- France
- Germany
- Italy
- Netherlands
- Romania
- Russia
- Saudi Arabia
- Spain
- Turkey
- UK
- USA

The caregivers each cared for one patient with AM (43 patients total).

The online survey was available from November 2022 to February 2023 in **13 different languages**.

Nov 2022 → Feb 2023

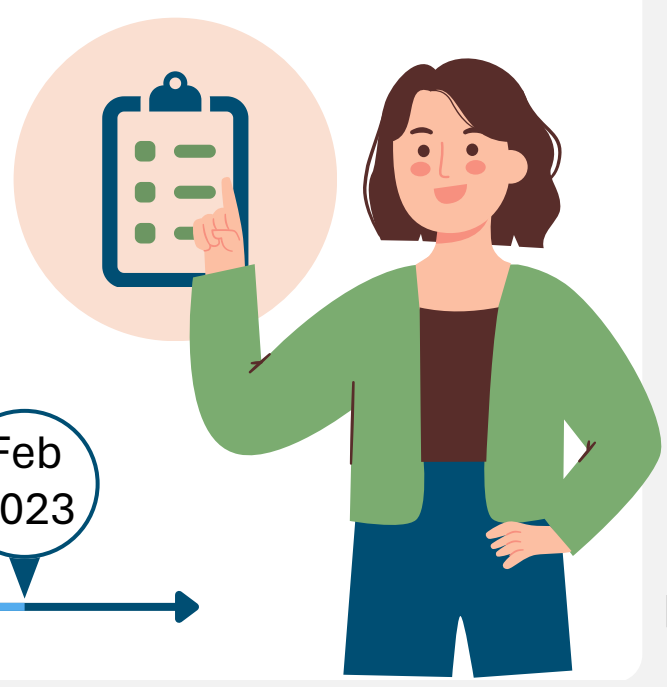

Patient details:

**16 patients** not treated	**6 patients** received HSCT	**21 patients** receiving ERT
Average **age** when **symptoms** were noticed: **6** years	Average **age** when **symptoms** were noticed: **3** years	Average **age** when **symptoms** were noticed: **3** years
	Average **age** when **treatment** started: **4** years	Average **age** when **treatment** started: **19** years
Average **age** at time of **survey**: **27** years	Average **age** at time of **survey**: **15** years	Average **age** at time of **survey**: **25** years

Caregiver details:

25% of caregivers of **not treated** people were **working***

50% of caregivers of people with **HSCT** were **working***

48% of caregivers of people on **ERT** were **working***

5 caregivers of **not treated** people said they were **retired**.

3 caregivers of people on **ERT** said they were **retired**.

*Full- or part-time.

What do these results mean for caregivers of people with AM?

The table below shows the average changes in VAS scores for caregivers who said their quality of life **improved**, **declined**, or **stayed the same** compared with 5 years ago.

Symbol	Meaning
+ +	improvement
+	slight improvement
=	no change
–	slight decline
– –	decline

Caregivers of...	Physical health	Mental health	Family & social life	Relationships	Ability to work/study
**Patients not treated** (16 caregivers)	slight decline	decline	decline	decline	slight decline
**Patients with HSCT** (6 caregivers)	slight improvement	improvement	improvement	slight improvement	slight improvement
**All patients on ERT** (21 caregivers)	slight improvement	no change	slight decline	slight improvement	slight improvement
**Children* on ERT** (4 caregivers)	no change	slight decline	slight improvement	slight improvement	slight improvement
**Adults* on ERT** (17 caregivers)	slight improvement	slight improvement	slight decline	slight improvement	slight improvement
**Patients on ERT for less than 5 years** (10 caregivers)	slight decline	slight decline	slight decline	slight decline	no change
**Patients on ERT for 5 years or more** (11 caregivers)	slight improvement	slight improvement	slight improvement	slight improvement	improvement

Classifications based on the differences in VAS scores from 5 years ago compared to the time of the survey, with differences of less than 1 classified as a slight change. *Children were under 16 years old and adults were at least 16 years old.

Caregivers of patients with HSCT and **on ERT for more than 5 years** had consistently **better** or **slightly better** quality of life after 5 years.

Caregivers of patients on ERT had **slight improvements** or no change in quality of life.

Slight declines in quality of life were seen across most areas for the caregivers of people who had been on ERT for **less than 5 years**. This might be because they were affected by the impacts of starting a new treatment and were feeling uncertain about how it might go.

Caregivers of not treated patients had consistent **declines** or **slight declines** in quality of life.

Although their scores declined over time, these caregivers had some of the best scores 5 years ago and at the time of the survey. This might be because the not treated patients were older and more independent than those who had treatment. Their caregivers would have had a longer time to get used to caring for someone with AM, and may also be retired.

What did the study tell us and why is it important?

1. This study gives valuable insights into the lives of people caring for those with AM, including the challenges they face. Over time, caregivers of people who received ERT or HSCT appear to have had a different quality of life experience, compared with caregivers of people who only received supportive care to manage AM symptoms.

2. It is important to consider caregiver experience as part of the overall impact of AM and make sure that caregivers get the support they need. More research is needed to better understand the various factors that affect quality of life for caregivers, including the severity of a person's AM and differences in healthcare and social support systems.

Points to consider: the findings should be viewed with caution. Although this study achieved a good overall level of participation for a rare disease, the small number of participants means we can only describe general patterns rather than draw firm conclusions. The quality of life of caregivers may also be influenced by factors like their own age, the patient's age, and how severe the disease is. The VAS measurement tool cannot pinpoint exact factors affecting quality of life and VAS responses might be influenced by cultural, regional and social or financial differences between participants.

THANK YOU!

The researchers would like to thank patients and caregivers for their time to participate in the survey and for sharing their experience with us to support this project to understand the progression of alpha-mannosidosis and how it affects caregivers' quality of life.

This summary was produced by Rare Disease Research Partners on behalf of Chiesi USA, Inc.
