# Supplementary material for: Quality of life and burden of caregivers of patients with alpha-mannosidosis: an international survey
Source: Orphanet J Rare Dis. 2026 Jul 22;21:385. doi: 10.1186/s13023-026-04496-y (PMC13732844; doi:10.1186/s13023-026-04496-y)
Supplement: Supplementary file 2 [file 13023_2026_4496_MOESM1_ESM.docx]

**Supplementary materials**

**Additional Table 1.** Mean VAS scores now and 5 years ago and mean change in VAS scores for caregivers’ physical health, mental health, family and social life, relationships and ability to work or attend education. Length of time on treatment varies for all patients irrespective of treatment group hence data from 5 years ago to now correspond to different follow up periods in relation to start of treatment.

| **Caregivers of** | | | | | | | | | | | | | |  |
| --- | --- | --- | --- | --- | --- | --- | --- | --- | --- | --- | --- | --- | --- | --- |
| **ERT patients** | | | | | | | | | | **HSCT patients** | | **UP** | |  |
| **CAREGIVERS VAS SCORES** | All | Pediatric <16 years old | | Adults ≥16 years old | On ERT  <5 years | On ERT  ≥5 years | Started ERT at pediatric age (<16 years old) | | Started ERT at adult age  (≥16 years old) | | All | | All | |
| N | 21 | | 4 | 17 | 10 | 11 | 9 | | 12 | | 6 | | 16 | |
| **Physical health** | | | |  |  |  |  | |  | |  | |  | |
| *5 years-ago* |  | |  |  |  |  |  | |  | |  | |  | |
| Median | 2.0 | | 1.5 | 3.0 | 4.0 | 2.0 | 1.0 | | 4.5 | | 2.5 | | 3.0 | |
| Mean ±SD | 3.9 ±3.3 | | 3.0 ±4.1 | 4.1 ±3.2 | 4.5 ±3.1 | 3.3 ±3.5 | 2.3 ±3.1 | | 5 ±3.1 | | 3.2 ±2.7 | | 2.5 ±2.4 | |
| Range | 0–10 | | 0–9 | 0–10 | 1–9 | 0–10 | 0–9 | | 0–10 | | 0–8 | | 0–6 | |
| *Now* |  | |  |  |  |  |  | |  | |  | |  | |
| Median | 2.0 | | 1.5 | 3.0 | 4.0 | 2.0 | 2.0 | | 5.0 | | 2.5 | | 4.0 | |
| Mean ±SD | 3.7 ±3.2 | | 3.0 ±4.1 | 3.9 ±3.1 | 4.6 ±3.0 | 2.9 ±3.3 | 2.7 ±3.4 | | 4.5 ±2.9 | | 3.0 ±2.6 | | 3.2 ±2.9 | |
| Range | 0–9 | | 0–9 | 0–9 | 1–9 | 0–9 | 0–9 | | 0–9 | | 0–7 | | 0–7 | |
| *Change in VAS scores* | | | | | | |  |  | |  | |  | |  |
| Median | 0.0 | | 0.0 | 0.0 | 0.0 | 0.0 | 0.0 | | 0.0 | | 0.0 | | 0.0 | |
| Mean ±SD | -0.1 ±2.8 | | 0.0 ±0.0 | -0.2 ±3.1 | 0.1 ±2.1 | -0.4 ±3.3 | 0.3 ±0.7 | | -0.5 ±3.6 | | -0.2 ±1.2 | | 0.7 ±0.9 | |
| Range | (-10)–4 | | 0–0 | (-10)–4 | (-4)–4 | (-10)–3 | 0–2 | | (-10)–4 | | (-2)–1 | | 0–3 | |
| **Mental health** | | |  |  |  |  |  | |  | |  | |  | |
| *5 years-ago* |  | |  |  |  |  |  | |  | |  | |  | |
| Median | 4.0 | | 2.5 | 5.0 | 5.5 | 2.0 | 3.0 | | 5.0 | | 8.0 | | 2.5 | |
| Mean ±SD | 4.1 ±2.9 | | 3.0 ±2.9 | 4.4 ±3.0 | 5.1 ±2.9 | 3.2 ±2.8 | 3.7 ±3.2 | | 4.4 ±2.9 | | 8.0 ±1.7 | | 3.2 ±3.3 | |
| Range | 0–10 | | 0–7 | 0–10 | 0–10 | 0–9 | 0–10 | | 0–9 | | 5–10 | | 0–9 | |
| *Now* |  | |  |  |  |  |  | |  | |  | |  | |
| Median | 4.0 | | 3.5 | 4.0 | 5.5 | 2.0 | 5.0 | | 4.0 | | 8.0 | | 5.0 | |
| Mean ±SD | 4.1 ±2.9 | | 3.3 ±2.8 | 4.3 ±3.0 | 5.3 ±3.0 | 3.0 ±2.4 | 4.4 ±3.4 | | 3.8 ±2.6 | | 7.0 ±3.6 | | 4.6 ±2.8 | |
| Range | 0–10 | | 0–6 | 0–10 | 0–10 | 0–6 | 0–10 | | 0–8 | | 0–10 | | 0–8 | |
| *Change in VAS scores* | | | | | | |  |  | |  | |  | |  |
| Median | 0.0 | | 0.0 | 0.0 | 0.0 | 0.0 | 0 | | 0 | | 0.0 | | 1.0 | |
| Mean ±SD | 0.0 ±2.3 | | 0.3 ±1.3 | -0.1 ±2.5 | 0.2 ±1.6 | -0.2 ±2.9 | 0.8 ( ±1.5) | | -0.6 ( ±2.7) | | -1.0 ±4.7 | | 1.4 ±1.9 | |
| Range | (-8)–3 | | (-1)–2 | (-8)–3 | (-3)–3 | (-8)–3 | (-1)–3 | | (-8)–3 | | (-10)–4 | | (-1)–5 | |
| **Family and social life** | | |  |  |  |  |  | |  | |  | |  | |
| *5 years-ago* |  | |  |  |  |  |  | |  | |  | |  | |
| Median | 3.0 | | 1.5 | 5.0 | 5.5 | 3.0 | 2.0 | | 6.0 | | 6.0 | | 2.5 | |
| Mean ±SD | 4.6 ±3.4 | | 2.3 ±2.6 | 5.2 ±3.4 | 5.4 ±3.0 | 3.9 ±3.7 | 3.0 ±3.5 | | 5.8 ±2.8 | | 6.5 ±1.8 | | 3.5 ±3.4 | |
| Range | 0–10 | | 0–6 | 0–10 | 1–10 | 0–10 | 0–10 | | 3–10 | | 4–9 | | 0–8 | |
| *Now* |  | |  |  |  |  |  | |  | |  | |  | |
| Median | 3.0 | | 1.5 | 6.0 | 6.5 | 2.0 | 2.0 | | 7.0 | | 6.5 | | 5.0 | |
| Mean ±SD | 4.7 ±3.6 | | 2.0 ±2.2 | 5.4 ±3.6 | 5.9 ±3.3 | 3.6 ±3.6 | 3.2 ±3.6 | | 5.8 ±3.2 | | 5.5 ±3.5 | | 4.6 ±3.7 | |
| Range | 0–10 | | 0–5 | 0–10 | 1–10 | 0–9 | 0–10 | | 1–10 | | 0–9 | | 0–10 | |
| *Change in VAS scores* | | | | | | |  |  | |  | |  | |  |
| Median | 0.0 | | 0.0 | 0.0 | 0.0 | 0.0 | 0.0 | | 0.5 | | -0.5 | | 1.0 | |
| Mean ±SD | 0.1 ±2.3 | | -0.3 ±0.5 | 0.2 ±.2.6 | 0.5 ±1.2 | -0.3 ±3.1 | 0.2 ( ±0.8) | | 0.0 ( ±3.1) | | -1.0 ±3.5 | | 1.1 ±1.4 | |
| Range | (-9)–3 | | (-1)–0 | (-9)–3 | (-1)–3 | (-9)–3 | (-1)–2 | | (-9)–3 | | (-6)–3 | | (-1)–5 | |
| **Relationships** |  | |  |  |  |  |  | |  | |  | |  | |
| *5 years-ago* |  | |  |  |  |  |  | |  | |  | |  | |
| Median | 3.0 | | 1.0 | 3.0 | 2 | 3 | 2.0 | | 3.5 | | 5.5 | | 2.0 | |
| Mean ±SD | 3.5 ±3.2 | | 2.0 ±2.8 | 3.9 ±3.3 | 2.8 ±2.9 | 4.2 ±3.6 | 2.7 ±2.9 | | 4.2 ±3.5 | | 5.2 ±3.4 | | 3.4 ±3.4 | |
| Range | 0–10 | | 0–6 | 0–10 | 0–8 | 0–10 | 0–8 | | 0–10 | | 0–9 | | 0–8 | |
| *Now* |  | |  |  |  |  |  | |  | |  | |  | |
| Median | 3.0 | | 1.5 | 4.0 | 3.5 | 3.0 | 2.0 | | 3.5 | | 4.5 | | 6.0 | |
| Mean ±SD | 3.3 ±2.7 | | 1.8 ±1.7 | 3.6 ±2.8 | 3.0 ±2.1 | 3.5 ±3.3 | 2.6 ±2.2 | | 3.8 ±3.0 | | 5.0 ±4.8 | | 4.7 ±3.6 | |
| Range | 0–9 | | 0–4 | 0–9 | 0–6 | 0–9 | 0–6 | | 0–9 | | 0–10 | | 0–10 | |
| *Change in VAS scores* | | | | | | |  |  | |  | |  | |  |
| Median | 0.0 | | 0.0 | 0.0 | 0.0 | 0.0 | 0 | | 0 | | 0.0 | | 1.0 | |
| Mean ±SD | -0.2 ±2.6 | | -0.3 ±1.3 | -0.2 ±2.9 | 0.2 ±1.9 | -0.6 ±3.2 | -0.1 ( ±1.3) | | –0.3 ( ±3.4) | | -0.2 ±2.1 | | 1.3 ±1.7 | |
| Range | (-10)–5 | | (-2)–1 | (-10)–5 | (-2)–5 | (-10)–2 | (-2)–2 | | (-10)–5 | | (-3)–3 | | (-1)–6 | |
| **Work/education** | | |  |  |  |  |  | |  | |  | |  | |
| *5 years-ago* |  | |  |  |  |  |  | |  | |  | |  | |
| Median | 5.0 | | 2.5 | 5.0 | 5.0 | 3.0 | 5.0 | | 4.0 | | 8.5 | | 1.5 | |
| Mean ±SD | 4.5 ±3.9 | | 2.8 ±3.2 | 4.9 ±4.0 | 4.7 ±3.7 | 4.4 ±4.2 | 4.4 ±4.1 | | 4.6 ±3.8 | | 6.8 ±4.1 | | 3.3 ±3.4 | |
| Range | 0–10 | | 0–6 | 0–10 | 0–10 | 0–10 | 0–10 | | 0–10 | | 0–10 | | 0–10 | |
| *Now* |  | |  |  |  |  |  | |  | |  | |  | |
| Median | 2.0 | | 1.5 | 2.0 | 5.0 | 2.0 | 3.0 | | 2.0 | | 7.0 | | 2.5 | |
| Mean ±SD | 4.0 ±3.9 | | 2.0 ±2.4 | 4.4 ±4.0 | 4.7 ±3.7 | 3.3 ±4.0 | 4.2 ±4.2 | | 3.8 ±3.8 | | 6.3 ±3.9 | | 4.1 ±4.1 | |
| Range | 0–10 | | 0–5 | 0–10 | 0–10 | 0–10 | 0–10 | | 0–10 | | 0–10 | | 0–10 | |
| *Change in VAS scores* | | | | | | |  |  | |  | |  | |  |
| Median | 0.0 | | -0.5 | 0.0 | 0.0 | 0.0 | 0 | | 0 | | 0.0 | | 1.0 | |
| Mean ±SD | -0.6 ±2.4 | | -0.8 ±1.0 | -0.5 ±2.6 | 0.0 ±1.2 | -1.1 ±3.0 | -0.2 ( ±1.5) | | -0.8 ( ±2.9) | | -0.5 ±1.8 | | 0.9 ±2.2 | |
| Range | (-10)–3 | | (-2)–0 | (-10)–3 | (-2)–3 | (-10)–1 | (-2)–3 | | (-10)–1 | | (-3)–2 | | (-1)–9 | |

**Additional Figure 1.** Caregivers’ individual changes in physical health.

**Additional Figure 1.** Individual changes in caregivers’ physical health VAS scores over 5 years within the three main treatment groups. Caregivers were asked to rate how caring for the person with alpha-mannosidosis had impacted their physical health now and 5-years ago on a VAS scale, where 0 was caring for the person with alpha-mannosidosis had no impact at all on my physical health and 10 caring for the person with alpha-mannosidosis has a severe impact on my physical health.

*ERT=enzyme replacement therapy; HSCT=hematopoietic stem cell transplantation; VAS=visual analog scale.*

- No change in physical health was reported for 9/16 (56.3%) caregivers of untreated patients while 7/16 (43.8%) experienced a worsening over time
- Equal numbers of caregivers of HSCT patients reported improvement, stabilization and worsening in physical health (for all, 2/6, 33.3%)
- Among caregivers of ET patients, physical health remained stable for 11/21 (52.4%), worsened for 7/21 (33.3%) and improved for 3/21 (14.3%)

**Additional Figure 2.** Caregivers’ individual changes in mental health.

**Additional Figure 2.** Individual changes in caregivers’ mental health VAS scores over 5 years within the three main treatment groups. Caregivers were asked to rate how caring for the person with alpha-mannosidosis had impacted their physical health now and 5-years ago on a VAS scale, where 0 was caring for the person with alpha-mannosidosis had no impact at all on my mental health and 10 caring for the person with alpha-mannosidosis has a severe impact on my mental health.

*ERT=enzyme replacement therapy; HSCT=hematopoietic stem cell transplantation; VAS=visual analog scale.*

- Mental health worsened in 10/16 (62.5%) of caregivers of untreated patients, stabilized in 4/16 (25.0%) and improved in 2/16 (12.5%)
- Equal numbers of caregivers of HSCT patients reported improvement, stabilization and worsening in mental health (for all, 2/6, 33.3%)
- Among caregivers of ERT patients, mental health remained stable in 11/21 (52.4%), worsened in 6/21 (28.6%) and improved in 4/21 (19.0%)
  - Of the 11 caregivers who reported that their mental health had remained the same, six and five were caring for patients treated for ≤6 years and ≥10 years, respectively
  - The three caregivers who reported a worsening in VAS score of +3 points cared for patients who started ERT at 11.3, 15.3 and 23.5 years and had been treated for 0.9, 5.8 and 5.9 years
  - The largest improvement in caregiver mental health VAS score (change in VAS score of –8 points) was reported by a caregiver for a patient who started ERT at 34.8 years and had been on treatment for 10.9 years

**Additional Figure 3.** Caregivers’ individual changes in family and social life.

**Additional Figure 3.** Individual changes in caregivers’ family and social life VAS scores over 5 years within the three main treatment groups. Caregivers were asked to rate how caring for the person with alpha-mannosidosis had impacted their family and social life now and 5-years ago on a VAS scale, where 0 was caring for the person with alpha-mannosidosis had no impact at all on my family and social life and 10 caring for the person with alpha-mannosidosis has a severe impact on my family and social life.

*ERT=enzyme replacement therapy; HSCT=hematopoietic stem cell transplantation; VAS=visual analog scale.*

- The majority of caregivers of untreated patients (9/16, 56.3%) reported a worsening in family and social life, while 6/16 (37.5%) and 1/16 (6.3%) reported a stabilization and improvement, respectively
- Half of caregivers of HSCT patients (3/6, 50%) experienced an improvement in family and social life, 1/6 (16.7%) a stabilization and 2/6 (33.3%) a worsening
- Among those caring for ERT patients, family and social life was stable in 10/21 (47.6%), worsened in 8/21 (38.1%) and improved over time for 3/21 (14.3%)
  - Of the 10 caregivers with stable family and social life, seven cared for patients on ERT for ≤6 years and three for patients on ERT for ≥10 years
  - Two caregivers reported an increase in VAS score of +3 points; these caregivers cared for patients who had started treatment at 23.5 and 37.5 years old and had been on ERT for 4.7 and 5.8 years
  - The caregiver with the largest improvement (change in VAS score of –9 points) cared for a patient who had received ERT for 10.9 years, starting at the age of 34.8 years old

**Additional Figure 4.** Caregivers’ individual changes in their relationship with partners, family or friends.

**Additional Figure 4.** Individual changes in caregivers’ relationship with partners, family or friends VAS scores over 5 years per treatment group. Caregivers were asked to rate how caring for the person with alpha-mannosidosis had impacted their relationships now and 5-years ago on a VAS scale, where 0 was caring for the person with alpha-mannosidosis had no impact at all on my relationships and 10 caring for the person with alpha-mannosidosis has a severe impact on my relationships.

*ERT=enzyme replacement therapy; HSCT=hematopoietic stem cell transplantation; VAS=visual analog scale.*

- Relationships worsened for 9/16 (56.3%) of caregivers of untreated patients, stabilized for 6/16 (37.5%) and improved for 1/16 (6.3%)
- Of the six caregivers of HSCT patients, two reported a worsening in relationships, two a stabilization and two an improvement (for all, 33.3%)
- For caregivers of ERT patients, relationships remained the same for 12/21 (57.1%), worsened for 5/21 (23.8%) and improved for 4/21 (19.0%) over the 5 years
  - Of the 12 caregivers who reported stable relationships, seven were caregivers of patients on ERT for ≤6 years and five cared for patients treated for ≥10 years
  - A worsening in VAS score of +5 points was reported by a caregiver of a patient who started treatment at 37.5 years and received treatment for 4.7 years; the largest improvement (change in score of −10 points) was for a caregiver of a patient on ERT for 10.9 years who started treatment at age 34.8 years

**Additional Figure 5.** Caregivers’ individual changes in their ability to work or attend education.

**Additional Figure 5.** Individual changes in caregivers’ ability to work/attend education VAS scores over 5 years per treatment group. Caregivers were asked to rate how caring for the person with alpha-mannosidosis had impacted their ability to work or attend education now and 5-years ago on a VAS scale, where 0 was caring for the person with alpha-mannosidosis had no impact at all on my ability to work or attend education and 10 caring for the person with alpha-mannosidosis has a severe impact on my ability to work or attend education.

*ERT=enzyme replacement therapy; HSCT=hematopoietic stem cell transplantation; VAS=visual analog scale.*

- Half of caregivers of untreated patients (8/16, 50.0%) had a stabilization in ability to work/attend education while 7/16 (43.8%) experienced worsening and 1/16 (6.3%) an improvement
- Two caregivers of HSCT patients reported an improvement (2/6, 33.3%) in the ability to work/attend education, while three reported no change (3/6, 50%) and one a worsening
- Ability to work/attend school remained the same for 14/21 (66.7%) caregivers of ERT patients, improved for 5/21 (23.8%) and worsened for the remaining two (9.5%) caregivers
  - Of the 14 caregivers whose ability to work or attend education did not change over 5 years, nine cared for patients who were on ERT for ≤6 years and five for patients who were on ERT for ≥10 years
  - A caregiver with a worsening in their ability to work or attend education of +3 cared for a patient who was on ERT for less than a year (0.9 years) having started in their second decade of life. The greatest improvement of –10 was reported for a caregiver of a patient who was on ERT for 10.9 years and started treatment in their fourth decade of life
